# Comparison of End-of-Life Care Between Recent Immigrants and Long-standing Residents in Ontario, Canada

**DOI:** 10.1001/jamanetworkopen.2021.32397

**Published:** 2021-11-02

**Authors:** Bradley I. Quach, Danial Qureshi, Robert Talarico, Amy T. Hsu, Peter Tanuseputro

**Affiliations:** 1 Faculty of Sciences, University of Ottawa, Ottawa, Canada; 2 Clinical Epidemiology Program, Ottawa Hospital Research Institute, Ottawa, Canada; 3 Bruyère Research Institute, Ottawa, Canada; 4 ICES, Ottawa, Canada; 5 Department of Family Medicine, Faculty of Medicine, University of Ottawa, Ottawa, Canada

## Abstract

**Importance:**

Recent immigrants face unique cultural and logistical challenges that differ from those of long-standing residents, which may influence the type of care they receive at the end of life.

**Objective:**

To compare places of care among recent immigrants and long-standing residents in Canada in the last 90 days of life.

**Design, Setting, and Participants:**

This population-based retrospective cohort study used linked health administrative data on individuals from Ontario, Canada, who died between January 1, 2013, and December 31, 2016, extracted on February 26, 2020. Individuals were categorized by immigration status: recent immigrants (since 1985) and long-standing residents. Data were analyzed from December 27, 2019, to February 26, 2020.

**Exposures:**

All decedents who immigrated to Canada between 1985 and 2016 were classified as recent immigrants. Subgroup analyses assessed the association of region of origin.

**Main Outcomes and Measures:**

The main outcome was place of care, including institutional and noninstitutional settings, in the last 90 days of life. Descriptive statistics were used to compare characteristics and health service utilization among recent immigrants and long-standing residents. Negative binomial regression models estimated the rate ratios (RR) of using acute care and long-term care in the last 90 days of life.

**Results:**

A total of 376 617 deceased individuals (median [IQR] age, 80 [68-88] years; 187 439 [49.8%] women and 189 178 [50.2%] men) were identified, among whom 22 423 (6.0%) were recent immigrants; recent immigrants were younger than long-standing residents (median [IQR] age, 76 [60-85] years vs 81 [69-88] years; *P* < .001), more likely to be living in lower income neighborhoods (12 357 immigrants [55.1%] vs 166 017 long-standing residents [46.9%] in the lower 2 income quintiles; *P* < .001), and had a higher Charlson Index score (score ≥5, 6294 immigrants [28.1%] vs 74 809 long-standing residents [21.1%]; *P* < .001). In the last 90 days of life, recent immigrants spent more days in intensive care units than long-standing residents (mean [SD], 2.64 [8.73] days vs 1.47 [5.70] days; *P* < .001), while long-standing residents spent more days using long-term care than recent immigrants (mean [SD], 19.49 [35.81] days vs 10.45 [27.42] days; *P* < .001). Being a recent immigrant was associated with a greater likelihood of acute inpatient care use (RR, 1.21; 95% CI, 1.18-1.24) and lower likelihood of long-term care use (RR, 0.66; 95% CI, 0.63-0.70), after adjusting for covariates.

**Conclusions and Relevance:**

These findings suggest that at the end of life, recent immigrants were significantly more likely to receive inpatient and intensive care unit services and die in acute care settings compared with long-standing residents. Further research is needed to examine differences in care preference and disparities for immigrant groups of different origins.

## Key Points

**Question:**

Is recent immigration status associated with place of care at the end of life compared with long-standing residents?

**Findings:**

In this cohort study of 376 617 deceased individuals in Ontario, Canada, recent immigrants who died were younger, had lower income and higher risk of mortality, and died more often in acute care settings compared with long-standing residents. In the last 90 days of life, recent immigrants were significantly more likely to receive emergency and acute (inpatient and/or intensive care unit services) care instead of long-term care than long-standing residents.

**Meaning:**

These findings suggest that at the end of life, recent immigrants were more likely to receive inpatient and intensive care unit services and die in acute care settings compared with long-standing residents, suggesting a need for more culturally appropriate care interventions for immigrants.

## Teaser

This cohort study examines places of care during the last 90 days of life among recent immigrants vs long-standing residents in Ontario, Canada.

## Introduction

In 2019, Canada welcomed more than 341 000 new permanent residents, where they represented 26% of the workforce and contributed more than 80% to population growth, and more than 50% had university degrees.^1^ However, despite their contribution to a productive and skilled Canadian society, immigrants can experience restricted health care, as they often face challenges, such as poverty, language difficulties, or policies that enforce delays on immigrants before they can receive health insurance.^2,3,4,5^ These barriers complicate the ability of immigrants to establish relationships with physicians, access prescriptions and medications, and seek appropriate health care. Such health care inequities may lead to greater odds of unmet health needs and health decline in immigrants compared with nonimmigrants.^6,7,8,9^

Death is an inevitable process for all; however, the level and quality of care at the end of life can differ drastically across individuals owing to unique cultural and ethnic practices.^10^ Regardless, individuals often share the same perspective on receiving personalized care and the preference to die at home.^11,12^ However, many patients continue to receive aggressive care near death and usually die in a hospital setting against their wishes.^13,14^ Population studies have found differences in patterns of care at the end of life among different ethnic groups, including higher use of intensive care units (ICUs), higher cost, and less hospice use among individuals who are members of ethnic minority groups, such as Asian and Hispanic groups.^15,16,17^ Despite these studies on ethnic and cultural differences in care, there is still a gap providing accessible, high-quality, culturally appropriate care to a diverse population.^12,16,17,18,19^ Few studies have considered immigrant status as a determinant of health associated with differences in care patterns or studied these trends across multiple care sectors. It is important to consider how health policies can be revised to provide patient-centric and culturally appropriate care that supports the increasing diversity of the population.^20,21,22^

To our knowledge, there are no population-based studies examining and comparing places of care across multiple settings at the end of life between recent immigrants and long-standing residents. To address this knowledge gap, we sought to compare the characteristics and place of care trajectories among recent immigrants and long-standing residents focused on the last 90 days of life.

## Methods

This cohort study uses data from ICES (formerly the Institute for Clinical Evaluative Sciences), an independent, nonprofit research institute whose legal status under Ontario’s health information privacy law allows it to collect and analyze health care and demographic data, without consent, for health system evaluation and improvement. The use of the data in this project is authorized under section 45 of Ontario’s Personal Health Information Protection Act and does not require review by a research ethics board. Our study followed the Strengthening the Reporting of Observational Studies in Epidemiology (STROBE) reporting guideline.

### Study Population

We conducted a population-based retrospective cohort study, capturing all residents aged 18 years or older who died in Ontario, Canada, between January 1, 2013, and December 31, 2016. Individuals were excluded if they were ineligible for provincial health insurance throughout the last year of life, had erroneous or missing data, had no record in the Vital Statistics Database (Office of the Registrar General–Deaths; ORGD), or were older than 105 years at time of death. Data were extracted on February 26, 2020.

Recent immigrants were identified using previous validated methods combining probabilistic and deterministic linkage of deceased individuals to the Immigration, Refugees and Citizenship Canada (IRCC) registry of landed immigrants.^23^ The IRCC database only captures landed immigrants from 1985 and onwards. Recent immigrants were defined as those granted permanent residency or citizenship in Canada between 1985 and 2016. All other individuals, such as those who landed prior to 1985 or those who were born in Canada, were defined as long-standing residents. Other studies have reserved the term *recent* for immigrants arriving within shorter timeframes, but our broad definition sought to include all available data and to acknowledge that individuals of the long-standing resident cohort may also be immigrants that have lived in Canada prior to 1985. IRCC data also includes country of birth and landing date, which was obtained at the time of application. Information on health literacy, religion, and specific cultural practices was unavailable. Countries of birth were organized in global regions following the United Nations method of regional classification (eMethods in the Supplement).^24^ Among recent immigrants and long-standing residents, we also analyzed trends by grouping decedents based on the following death trajectories, using the *International Statistical Classification of Diseases and Related Health Problems, Tenth Revision, Canada* (*ICD-10-CA*) codes from death certificates as per previous methods, including frailty, organ failure, terminal illness, sudden death, or other.^25,26^

### Data Sources and Study Definition

We used unique encoded identifiers to link multiple health administrative databases held and analyzed at ICES. These data sets contain deidentified health information for all Ontario residents and comprehensively track all health care encounters for medically necessary services funded by the provincial government. The following databases were used in this study: ORGD for capturing place, cause, date of death, and demographic information; Ontario Health Insurance Plan Claims Database for physician-based services in inpatient and outpatient settings; Home Care Database for publicly funded home care services; Discharge Abstract Database for capturing all hospitalization data; National Ambulatory Care Reporting System for all emergency department (ED) visits; National Rehabilitation Reporting System for inpatient rehabilitation programs; Continuing Care Reporting System for long-term and complex continuing care services; Statistics Canada Census data for income quintile and community size; and IRCC Permanent Resident Database for permanent residency or citizenship status for individuals starting from 1985. In Ontario and across Canada, recent immigrants have coverage to free emergency services immediately after arrival. In some provinces, there is a waiting period of up to 3 months for other health services covered under the universal health care system, although Ontario has removed this waiting period. The Ontario Health Insurance Plan provides eligible persons with a Provincial Health Card that enables their access to and the tracking of data on all services and procedures provided that resulted in charges to the health care system.^27^

### Outcomes

The primary outcome was each patient’s places of care in the last 90 days of life among recent immigrants compared with long-standing residents. We captured the following places of care, adopting a hierarchy for service use (to control for any overlap of sector use on any given day): ICU stay, non-ICU hospital admissions, ED care, complex continuing care, rehabilitation services, long-term care, and home care. If an individual encounters multiple places of care for any given day, the day is attributed to service holding the highest priority from the preceding list. We also examined the location of death in hospital and community settings. For our regression analyses, the outcomes on which we assessed the association of immigrant status and covariates were the rates of long-term care and inpatient care in the last 90 days of life.

### Statistical Analysis

We used descriptive and inferential statistics to describe and contrast baseline characteristics and places of care between recent immigrants and long-standing residents. Characteristics included age, sex, income quintile, community size, chronic conditions (eTable 1 in the Supplement), Charlson Comorbidity Index, receipt of home care and palliative physician services in the last 90 days of life, and the cause of death trajectory. In addition, we examined recent immigrant characteristics, such as region of origin and time since immigration (eTable 2, eTable 3, eFigure 1, and eFigure 2 in the Supplement). Line graphs were used to project the place of care trajectories for recent immigrants and long-standing residents; specifically, we reported the rate of deceased individuals who used a particular service per 100 000 deceased individuals on each day for the last 90 days of life. Using the same method, we also examined the hospital inpatient service use trends stratified by the cause of death between groups.

We conducted negative binomial regression analyses to assess factors associated with use of acute inpatient care in the last 90 days of life and use of long-term care in the last 90 days of life. All analyses were adjusted for potential confounders using immigrant status and baseline characteristics as covariates. All analyses were performed with SAS Enterprise Guide version 9.4 (SAS Institute). *P* values were 2-sided, and statistical significance was set at 95%. Data were analyzed from December 27, 2019, to February 26, 2020.

## Results

### Demographic Characteristics

We identified a total of 376 617 deceased individuals (median [IQR] age, 80 [68-88] years; 187 439 [49.8%] women and 189 178 [50.2%] men), of whom 22 423 (6.0%) immigrated since 1985; recent immigrants, compared with long-standing residents, were younger (median [IQR] age, 76 [60-85] years vs 81 [69-88] years; *P* < .001), in lower income quintiles (12 357 immigrants [55.1%] vs 166 017 [46.9%] long-standing residents in the lower 2 income quintiles; *P* < .001), and had a higher Charlson Index score (score ≥5, 6294 immigrants [28.1%] vs 74 809 long-standing residents [21.1%]; *P* < .001) (Table 1). In most settings, recent immigrants also received more palliative physician visits than long-standing residents, particularly in outpatient care (8986 immigrants [40.1%] vs 113 797 long-standing residents [32.1%]; *P* < .001) and third party settings (8908 immigrants [39.7%] vs 109 126 long-standing residents [30.%]; *P* < .001). Compared with long-standing residents, recent immigrants were more likely to have diabetes (131 503 long-standing residents [37.1%] vs 9793 immigrants [43.7%]; *P* < .001) and less likely to have chronic obstructive pulmonary disease (90 556 long-standing residents [25.6%] vs 3211 immigrants [14.3%]; *P* < .001), coronary disease (138 643 long-standing residents [39.1%] vs 6494 immigrants [29.0%]; *P* < .001), and osteoarthritis (239 150 long-standing residents [67.5%] vs 12 393 immigrants [55.3%]; *P* < .001). Immigrants were more likely to die of organ failure (8544 individuals [38.1%]) and terminal illness (7411 individuals [33.1%]) than frailty (3705 individuals [16.5%]) (*P* < .001). For long-standing residents, more died owing to organ failure (149 363 individuals [42.2%]) compared with terminal illness (99 641 individuals [28.1%]) and frailty (66 957 individuals [18.9%]) (*P* < .001). Recent immigrants originated from diverse global regions (eMethods in the Supplement).

**Table 1.** Baseline Characteristics of Decedents Among Recent Immigrants and Long-standing Residents

Characteristic	No. (%)			*P* value
	Long-standing residents (n = 354 194)	Recent immigrants (n = 22 423)	Total (N = 376 617)	
Age at death, y				
Median (IQR)	81 (69-88)	76 (60-85)	80 (68-88)	<.001
18-44	178 (0.1)	20 (0.1)	198 (0.1)	<.001
45-64	10 455 (3.0)	1591 (7.1)	12 046 (3.2)	
65-84	54 064 (15.3)	5416 (24.2)	59 480 (15.8)	
≥85	154 627 (43.7)	9161 (40.9)	163 788 (43.5)	
Sex				
Women	176 376 (49.8)	11 063 (49.3)	187 439 (49.8)	.183
Men	177 818 (50.2)	11 360 (50.7)	189 178 (50.2)	
Income quintile				
Lowest	88 902 (25.1)	7294 (32.5)	96 196 (25.5)	<.001
Low	77 115 (21.8)	5063 (22.6)	82 178 (21.8)	
Middle	67 387 (19.0)	4241 (18.9)	71 628 (19.0)	
High	61 217 (17.3)	3312 (14.8)	64 529 (17.1)	
Highest	58 189 (16.4)	2442 (10.9)	60 631 (16.1)	
Community size, No.				
≥1 500 000	105 668 (29.8)	17 326 (77.3)	122 994 (32.7)	<.001
500 000-1 499 999	59 408 (16.8)	2375 (10.6)	61 783 (16.4)	
100 000-499 999	93 926 (26.5)	1905 (8.5)	95 831 (25.4)	
10 000-99 999	42 090 (11.9)	299 (1.3)	42 389 (11.3)	
<10 000	51 202 (14.5)	339 (1.5)	51 541 (13.7)	
Charlson Comorbidity Index score				
0	141 434 (39.9)	7211 (32.2)	148 645 (39.5)	<.001
1-2	85 618 (24.2)	5229 (23.3)	90 847 (24.1)	
3-4	52 333 (14.8)	3689 (16.5)	56 022 (14.9)	
≥5	74 809 (21.1)	6294 (28.1)	81 103 (21.5)	
Cause of death				
Frailty	66 957 (18.9)	3705 (16.5)	70 662 (18.8)	<.001
Organ failure	149 363 (42.2)	8544 (38.1)	157 907 (41.9)	
Terminal illness	99 641 (28.1)	7411 (33.1)	107 052 (28.4)	
Sudden death	22 864 (6.5)	1798 (8.0)	24 662 (6.5)	
Other	14 768 (4.2)	949 (4.2)	15 717 (4.2)	
Receipt of home care in the last 90 d of life				
No home care	165 478 (46.7)	10 215 (45.6)	175 693 (46.7)	<.001
Nonpalliative home care	118 448 (33.4)	7337 (32.7)	125 785 (33.4)	
Palliative home care	70 268 (19.8)	4871 (21.7)	75 139 (20.0)	
Receipt of palliative care from a physician in the last 90 d of life across different settings				
Outpatient	113 797 (32.1)	8986 (40.1)	122 783 (32.6)	<.001
Inpatient stay	62 063 (17.5)	5317 (23.7)	67 380 (17.9)	<.001
Complex continuing care stay	7972 (2.3)	325 (1.4)	8297 (2.2)	<.001
Third party billing^a^	109 126 (30.8)	8908 (39.7)	118 034 (31.3)	<.001
Home visit	72 250 (20.4)	4718 (21.0)	76 968 (20.4)	.02
Prevalent conditions^b^				
Arrhythmia	87 830 (24.8)	4122 (18.4)	91 952 (24.4)	<.001
Chronic obstructive pulmonary disease	90 556 (25.6)	3211 (14.3)	93 767 (24.9)	<.001
Coronary disease	138 643 (39.1)	6494 (29.0)	145 137 (38.5)	<.001
Dementia	108 094 (30.5)	5198 (23.2)	113 292 (30.1)	<.001
Diabetes	131 503 (37.1)	9793 (43.7)	141 296 (37.5)	<.001
Osteoarthritis	239 150 (67.5)	12 393 (55.3)	251 543 (66.8)	<.001

^a^ Third party visits include consultations and care provided over telephone and case conferences.

^b^ Top 6 major prevalent conditions by percentage difference are listed; other prevalent conditions can be found in eTable 3 in the Supplement.

### Places of Care at the End of Life and Location of Death

Table 2 reports the mean number of days spent in each care setting in the last 90 days of life, as well as the location of death among recent immigrants and long-standing residents. In the last 90 days of life, recent immigrants made greater use of acute care than long-standing residents, spending more days in ICUs (mean [SD], 2.64 [8.73] days vs 1.47 [5.70] days; *P* < .001), non-ICU acute care (mean [SD], 11.91 [16.88] days vs 10.12 [15.72] days; *P* < .001), and EDs (mean [SD], 0.74 [1.06] days vs 0.71 [1.14] days; *P* < .001). In contrast, long-standing residents made greater use of subacute and long-term care services, spending more days in complex continuing care (mean [SD], 2.83 [12.67] days vs 2.58 [12.05] days; *P* < .001), rehabilitation (mean [SD], 0.42 [3.69] days vs 0.29 [2.97] days; *P* < .001), and long-term care (mean [SD], 19.49 [35.81] days vs 10.45 [27.43] days, *P* < .001). Of 376 617 deceased individuals, 172 361 (45.8%) died in acute care settings. Compared with long-standing residents, recent immigrants died more often in acute care settings (159 688 long-standing residents [45.1%] vs 12 674 immigrants [56.5%]; *P* < .001) but less often in long-term care settings (63 671 long-standing residents [18.0%] vs 1780 immigrants [7.9%]; *P* < .001). Among recent immigrants, individuals from Southern Europe (mean [SD], 13.3 [30.6] days; *P* < .001), Northern and Western Europe (mean [SD], 18.2 [35.2] days; *P* < .001), and East Asia (mean [SD], 17.3 [33.6] days; *P* < .001) spent more time in long-term care compared with individuals from other regions, while individuals from Northern and Western Europe (mean [SD] 9.2 [14.5] days; *P* < .001) spent less time in hospital care (eFigure 1 in the Supplement).

**Table 2.** Location of Death and Mean Number of Days Spent in Different Places of Care in the Last 90 Days of Life for Deceased Individuals in Ontario, Canada

Characteristic	Long-standing residents (n = 354 194)	Recent immigrants (n = 22 423)	Total (n = 376 617)	*P* value
Time receiving health care service in different locations in the last 90 d of life, mean (SD), d^a^				
ICU	1.47 (5.70)	2.64 (8.73)	1.54 (5.93)	<.001
Acute care hospitalization (non-ICU)	10.12 (15.72)	11.91 (16.88)	10.23 (15.79)	<.001
Emergency department	0.71 (1.14)	0.74 (1.06)	0.72 (1.13)	<.001
Long-term care home	19.49 (35.81)	10.45 (27.43)	18.95 (35.43)	<.001
Complex continuing care	2.83 (12.67)	2.58 (12.05)	2.82 (12.63)	.005
Home care (eg, physician home visits, personal support worker, nursing, physiotherapy)	13.22 (22.56)	15.33 (24.45)	13.35 (22.69)	<.001
Rehabilitation	0.42 (3.69)	0.29 (2.97)	0.42 (3.65)	<.001
Location of death, No. (%)^b^				
Acute care hospital	159 688 (45.1)	12 674 (56.5)	172 362 (45.8)	<.001
Complex continuing care hospital	29 328 (8.3)	1780 (7.9)	31 108 (8.3)	
Long-term care	63 671 (18.0)	1767 (7.9)	65 438 (17.4)	
Community	101 507 (28.7)	6202 (27.7)	107 709 (28.6)	

Abbreviation: ICU, intensive care unit.

^a^ Each mean number of days in care service reported *P* < .005.

^b^ Location of death settings are defined as: acute care hospital: ICU hospitalization, non-ICU hospitalization, emergency department care; complex continuing care: subacute care setting, rehabilitation; long-term care: long-term care; and community: home care, death at home.

### Place of Care Trajectories

**Figure 1.**
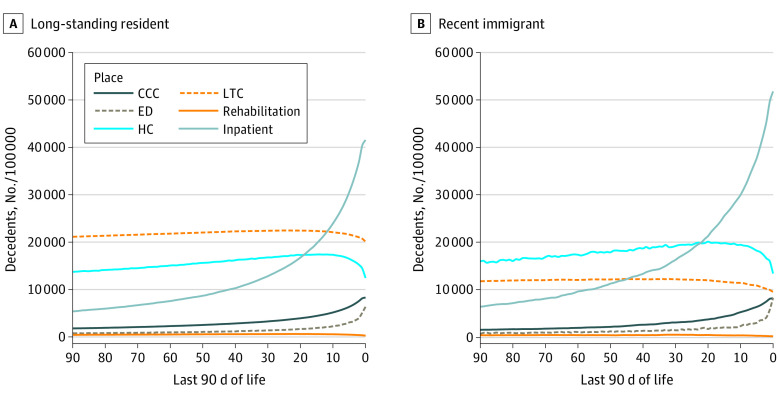
Comparison of the Places of Care in the Last 90 Days of Life Among Recent Immigrants and Long-standing Residents CCC indicates complex continuing care; ED, emergency department; HC, home care; LTC, long-term care; and inpatient, intensive care unit and non–intensive care unit hospitalization.

Figure 1 displays the place of care trajectories in the last 90 days of life among long-standing residents and recent immigrants, specifically describing ICU hospitalization, non-ICU hospitalization, ED care, complex continuing care, rehabilitation services, long-term care, and home care over time. Use of inpatient (ICU and non-ICU) services, ED, and complex continuing care services consistently increased as death became closer. Notably, use of inpatient care and ED care increased rapidly in the last 30 days of life, with the increase in use being greater among recent immigrants. Use of rehabilitation services remained relatively steady across the entire duration of the last 90 days of life among recent immigrants and long-standing residents. As death approached, use of home care services steadily increased among both groups, with a rapid decline in use particularly in the last week of life. Compared with recent immigrants, long-standing residents made greater use of long-term care services throughout the entire duration of the last 90 days of life. Among recent immigrants, acute care use also varied by region of origin with Asian (East, Southeast, Western, and Central), African, and South American immigrants typically using more acute care than European (Northern and Western) and North American immigrants (eFigure 2 in the Supplement).

**Figure 2.**
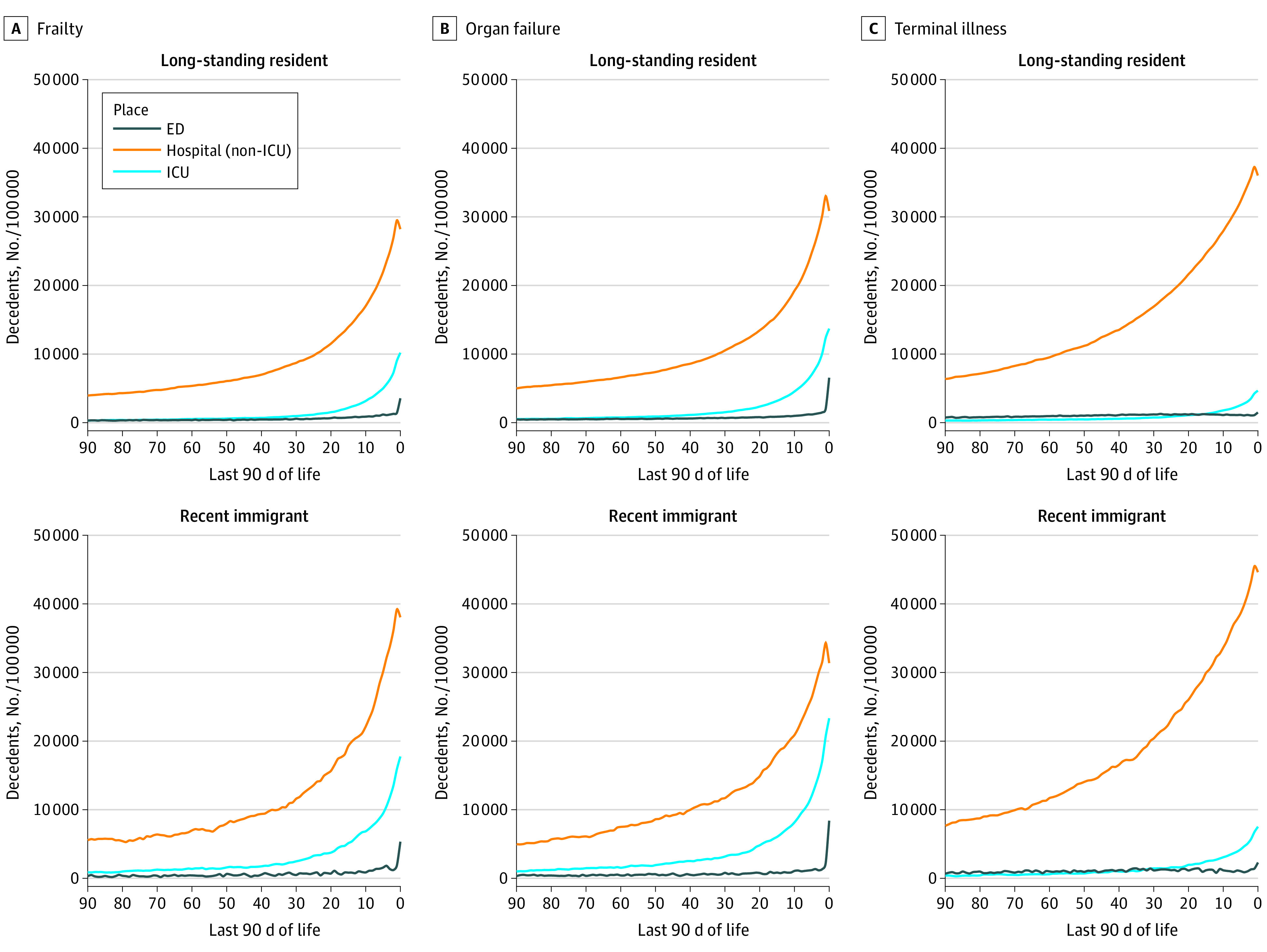
Comparison of the Places of Care in the Last 90 Days Among Recent Immigrants and Long-standing Residents Stratified by the Cause of Death ED indicates emergency department care; ICU, intensive care unit.

We also investigated acute care use trajectories among recent immigrants and long-standing residents, stratified by cause of death (frailty, organ failure, or terminal illness) (Figure 2). Among all groups, non-ICU inpatient care was the most used service throughout the last 90 days of life, with its use increasing rapidly as death became closer. Notably, individuals with terminal illness exhibited the greatest increase in the use of non-ICU inpatient care, particularly in the last 30 days of life, with recent immigrants displaying greater rates of increase than long-standing residents. Individuals who died from frailty and organ failure used more ICU and ED care services in the last week of life, with recent immigrants displaying greater rates of increase compared with long-standing residents.

### Multivariable Regression Analyses

In Table 3, our negative binomial regression analysis evaluated the likelihood of inpatient and long-term care service use in the last 90 days of life, adjusting for key covariates. When we examined the rate ratios (RRs) of using acute inpatient services in the last 90 days of life, being a recent immigrant was associated with greater rates of using inpatient care (RR, 1.21; 95% CI, 1.18-1.24) compared with long-standing residents after adjusting for key covariates. Individuals who died aged 85 years or older had lower rates of inpatient care (RR, 0.65; 95% CI, 0.64-0.67) compared with individuals who died aged 45 to 64 years. Compared with individuals who died of frailty, there were greater rates in inpatient use from deaths due to terminal illness (RR, 1.74; 95% CI, 1.71-1.77). An increasing Charlson comorbidity index score was associated with greater rates of inpatient use (score ≥5: RR, 7.03; 95% CI, 6.72-7.36) and greater rates of long-term care use (score ≥5: RR, 4.88; 95% CI, 4.38-5.44). For long-term care, being a recent immigrant was associated with lower rates of use (RR, 0.66; 95% CI, 0.63-0.70) compared with long-standing residents. Decedents who died aged 85 years or older had greater rates of long-term care (RR, 8.13; 95% CI, 7.80-8.49) compared with individuals who died aged 45 to 64 years.

**Table 3.** Negative Binomial Regression Models of Total Number of Inpatient and Long-term Care Days in the Last 90 Days of Life for Ontario Decedents

Exposure	Count of acute care hospital (ICU and non-ICU) days, rate ratio (95% CI)	*P* value	Count of long-term care days, rate ratio (95% CI)	*P* value
Sex				
Women	1 [Reference]	<.001	1 [Reference]	<.001
Men	1.10 (1.09-1.11)		0.68 (0.66-0.70)	
Age at death, y				
18-44	0.88 (0.85-0.91)	<.001	0.21 (0.20-0.23)	<.001
45-64	1 [Reference]	NA	1 [Reference]	NA
65-84	0.92 (0.91-0.94)	<.001	3.69 (3.54-3.84)	<.001
≥85	0.65 (0.64-0.67)	<.001	8.13 (7.80-8.49)	<.001
Charlson Comorbidity Index score				
0	1 [Reference]	NA	1 [Reference]	NA
1-2	3.56 (3.40-3.73)	<.001	3.00 (2.69-3.35)	<.001
3-4	4.93 (4.71-5.15)	<.001	3.94 (3.53-4.39)	<.001
≥5	7.03 (6.72-7.36)	<.001	4.88 (4.38-5.44)	<.001
Income quintile				
First (lowest)	1 [Reference]	NA	1 [Reference]	NA
Second	1.02 (1.00-1.04)	.009	0.88 (0.84-0.91)	<.001
Third	1.00 (0.98-1.02)	.98	0.87 (0.84-0.91)	<.001
Fourth	0.99 (0.97-1.00)	.13	0.91 (0.88-0.95)	<.001
Fifth (highest)	0.99 (0.99-1.01)	.22	0.83 (0.80-0.86)	<.001
Community size, No.				
≥1 500 000	1.13 (1.11-1.15)	<.001	0.69 (0.66-0.72)	<.001
500 000-1 499 999	0.97 (0.96-0.99)	.01	0.92 (0.88-0.97)	.001
100 000-499 999	0.93 (0.91-0.95)	<.001	0.90 (0.86-0.94)	<.001
10 000-99 999	0.94 (0.92-0.96)	<.001	1.04 (0.98-1.09)	.17
<10 000	1 [Reference]	NA	1 [Reference]	NA
Cause of death				
Frailty	1 [Reference]	NA	1 [Reference]	NA
Organ failure	1.16 (1.15-1.18)	<.001	0.44 (0.42-0.46)	<.001
Terminal illness	1.74 (1.71-1.77)	<.001	0.16 (0.16-0.17)	<.001
Sudden death	0.94 (0.91-0.96)	<.001	0.25 (0.23-0.27)	<.001
Other	1.47 (1.42-1.51)	<.001	0.57 (0.53-0.61)	<.001
Receipt of home care in the last 90 d of life				
No home care	1 [Reference]	NA	1 [Reference]	NA
Nonpalliative home care	1.59 (1.57-1.61)	<.001	0.81 (0.79-0.84)	<.001
Palliative home care	0.93 (0.91-0.95)	<.001	0.29 (0.27-0.30)	<.001
Receipt of palliative physician visit in the last 90 d of life	0.90 (0.89-0.92)	<.001	1.02 (0.98-1.06)	.37
Immigrant status				
Yes	1.21 (1.18-1.24)	<.001	0.66 (0.63-0.70)	<.001
No	1 [Reference]		1 [Reference]	

Abbreviations: ICU, intensive care unit; NA, not applicable.

## Discussion

In this population-based cohort study of Ontario residents who died between 2013 and 2016, we found that recent immigrants were 1.2-fold more likely to use acute care services at the end of life and 1.1-fold more likely to die in acute care settings compared with long-standing residents. In the last 90 days of life, despite a greater percentage of recent immigrants receiving palliative physician services (0.6%-8.9% more, depending on setting), recent immigrants spent a mean of 1.1 to 1.8 more days using inpatient care services (ICU and non-ICU) and ED services. More than half of recent immigrants died in hospital settings, compared with 45% of long-standing residents.

The variation in places of care observed in this study could be partially attributed to immigrant status, influenced by the region of origin and time since immigration. The disproportionate use of acute care among immigrants is corroborated by a previous study that found that Canadian immigrants were significantly more likely to receive aggressive care and die in an ICU compared with long-standing residents.^28^

Even after adjusting for covariates, we found that immigrant status was still a risk factor associated with increased likelihood of using inpatient services compared with long-term services. Studies in North America that examined health care utilization between immigrants and nonimmigrants support these findings, as immigrants reported greater difficulty in accessing care, leading to greater use of acute care and burdensome, life-sustaining interventions compared with nonimmigrants, who were less likely to report difficulties in care and used more end-of-life specific services such as hospice care.^8,9,15,16,17^

The high acute care use by recent immigrants may be associated with patient preferences, cultural differences, and care access. Qualitative research across multiple cultural settings has identified differences in the end-of-life care provided that are associated with the region of origin.^29,30,31,32^ Within Asia and Europe, variations in the level of aggressive, life-prolonging interventions are associated with the region, culture, and religion of patients and physicians.^20,33,34^ Studies on Canadian and American populations report similar care preferences within their respective ethnic cultures.^3,22,35,36,37^ The delivery of care is also critical to the end-of-life experience, as clinician approaches may differ by their sociocultural practices; patient and physician interests may be misaligned and therefore create a barrier for immigrants to receive culturally appropriate care.^38^ Discussions to initiate end-of-life and palliative care may improve the quality of life but can conflict with family decision-making processes guided by various cultural practices.^39,40^ The differences in end-of-life care may attenuate with time since immigration, as individuals become more accultured and familiar with the host system.^41^

The tendency for decedents to receive costly acute care at the end of life places a significant burden on the health care system.^42,43,44^ Providing acute care when community services may be cheaper and more aligned with patient preferences increases the strain on health care systems that are already facing issues with capacity issues in acute care, long wait times, and increasing caregiver stress and physician burnout.^45^ Restrictive policies and communication difficulties that exclude immigrants from receiving health care coverage and health literacy may exacerbate the burden, leading to a reliance on acute care among immigrants.^42^ However, our findings did not find associations by the community size or by sudden death or other cause of death.

### Limitations

Our study has several limitations. Capturing palliative care using administrative health data includes the potential undercoding of palliative care use received owing to potential palliative care services or approaches that are provided within another care element or setting but are not billed and recorded as palliative care. Second, the IRCC database only captures immigrant data from 1985 and onwards, therefore all immigrants who arrived before 1985 are classified as long-standing residents. However, previous research and our supplemental analysis has shown that with increasing time since immigration, the health profiles of immigrants typically resemble those of individuals born in Canada.^46^ There were no data or analyses of marital status, language ability for long-standing residents, education level for long-standing residents, goals of care, quality of care, and preferences for any patients or families, which limits our comparison between recent immigrants and long-standing residents and interpretation on whether individuals received sufficient, quality care to their preferences. Deceased individuals categorized into disease trajectories may overgeneralize results, since the health trajectory for each of the groupings may not follow the traditional pattern of health owing to other conditions, including comorbidities and the quality of care provided by caregivers. The administrative health data sets do not include and cannot account for services provided in a residential hospice. However, death rates in hospices are relatively low in Canada, and most hospice care follows initiation of home care services, which is included in our study.^47^

## Conclusions

In this cohort study among individuals who died in Ontario between 2013 and 2016, recent immigrants were more likely to use aggressive health services toward the end of life and die in acute care settings compared with long-standing residents. Future studies should investigate the associations of patient preferences, accessibility to end-of-life services, and comorbidities to acute care use in immigrant populations to direct resources for more effective end-of-life care.

## Notes

Supplement.
eMethods.
**eTable 1.** Comorbidities for Recent Immigrants and Long-standing Residents **eTable 2.** Location of Death for Recent Immigrants by Region of Origin and Time Since Immigration **eTable 3.** Negative Binomial Regression for Inpatient Care and Long-term Care Days in the Last 90 Days of Life for the Cohort of Recent Immigrants **eFigure 1.** Mean Number of Days Spent in Each Place of Care in the Last 90 Days of Life Among Recent Immigrants by Region of Origin **eFigure 2.** Comparison of Places of Care in the Last 90 Days of Life Among Recent Immigrants
