# Supplementary material for: Comparison of End-of-Life Care Between Recent Immigrants and Long-standing Residents in Ontario, Canada
Source: JAMA Netw Open. 2021 Nov 2;4(11):e2132397. doi: 10.1001/jamanetworkopen.2021.32397 (PMC8564577; doi:10.1001/jamanetworkopen.2021.32397)
Supplement: Supplement. — eMethods. eTable 1. Comorbidities for Recent Immigrants and Long-standing Residents eTable 2. Location of Death for Recent Immigrants by Region of Origin and Time Since Immigration eTable 3. Negative Binomial Regression for Inpatient Care and Long-term Care Days in the Last 90 Days of Life for the Cohort of Recent Immigrants eFigure 1. Mean Number of Days Spent in Each Place of Care in the Last 90 Days of Life Among Recent Immigrants by Region of Origin eFigure 2. Comparison of Places of Care in the Last 90 Days of Life Among Recent Immigrants [file jamanetwopen-e2132397-s001.pdf]

Supplemental Online Content

Quach BI, Qureshi D, Talarico R, Hsu AT, Tanuseputro P. Comparison of end-of-life care between recent immigrants and long-standing residents in Ontario, Canada. *JAMA Netw Open*. 2021;4(11):e2132397. doi:10.1001/jamanetworkopen.2021.32397

eMethods.
eTable 1. Comorbidities for Recent Immigrants and Long-standing Residents
eTable 2. Location of Death for Recent Immigrants by Region of Origin and Time Since Immigration
eTable 3. Negative Binomial Regression for Inpatient Care and Long-term Care Days in the Last 90 Days of Life for the Cohort of Recent Immigrants
eFigure 1. Mean Number of Days Spent in Each Place of Care in the Last 90 Days of Life Among Recent Immigrants by Region of Origin
eFigure 2. Comparison of Places of Care in the Last 90 Days of Life Among Recent Immigrants

This supplemental material has been provided by the authors to give readers additional information about their work.

© 2021 Quach BI et al. *JAMA Network Open.*

eMethods

Basic Definitions

Study inclusion - all individuals who died in Ontario between January 1, 2013 and December 31, 2016, excluding individuals with less than 12 months of enrollment in the provincial health care plan, individuals younger than 18 or older than 105 years of age.

Recent immigrant - an individual in the study population granted permanent residency status or citizenship in Canada between 1985 and 2016 according to the Immigration, Refugees, and Citizenship Canada data.

Long-standing resident - an individual included in the study who is not a recent immigrant.

Analysis Plan / Protocol

1) Primary analysis - compare the places of care and location of death for recent immigrant and long-standing resident cohorts in multiple outcome analyses (intensive-care unit hospitalization, non-intensive-care unit hospitalization, emergency department care, complex continuing care, rehabilitation services, long-term care, and home care delivery).

2) Primary subgroup analysis - repeat the primary analysis on the places of care and location of death focused on acute care services (intensive-care unit hospitalization, non-intensive care unit hospitalization, and emergency department care) stratified by cause of death. Results displayed by line graph.

3) Primary adjusted analysis - analyze days of acute inpatient care and long-term care across both cohorts using negative binomial regression to assess for the influence of immigration status, sex, age, Charlson comorbidity infex, income quintile, community size, cause of death, receipt of homecare between 90 to 365 days of life, and receipt of palliative physician vists between 90 to 365 days of life.

4) Primary analysis reported within recent immigrant subgroup - repeat the primary analysis on the days of acute inpatient care and long-term care across the recent immigrant cohort to assess the influence of time since immigration and region of origin in addition to sex, age, Charlson comorbidity infex, income quintile, community size, cause of death, receipt of homecare between 90 to 365 days of life, and receipt of palliative physician vists between 90 to 365 days of life.

5) Secondary analysis - compare the mean number of days at each place of care in the last 90 days of life and the location of death within the recent immigrant cohorts with respect to the region of origin and time since immigration.

Regional Definitions

Drawn from the United Nations at https://unstats.un.org/unsd/methodology/m49/overview/.

Northern Europe and Western Europe - Åland Islands, Channel Islands, Denmark, Estonia, Faeroe Islands, Finland, Guernsey, Iceland, Ireland, Isle of Man, Jersey, Latvia, Lithuania, Norway, Sark, Svalbard and Jan Mayen Islands, Sweden, United Kingdom of Great Britain and Northern Ireland, Austria, Belgium, France, Germany, Liechtenstein, Luxembourg, Monaco, Netherlands, Switzerland

Southern Europe - Albania, Andorra, Bosnia and Herzegovina, Croatia, Gibraltar, Greece, Holy See, Italy, Malta, Montenegro, Portugal, San Marino, Serbia, Slovenia, Spain, The former Yugoslav Republic of Macedonia, Eastern Europe: Belarus, Bulgaria, Czech Republic, Hungary, Poland, Republic of Moldova, Romania, Russian Federation, Slovakia, Ukraine

© 2021 Quach BI et al. *JAMA Network Open.*

Western and Central Asia - Afghanistan, Iran, Armenia, Azerbaijan, Bahrain, Cyprus, Georgia, Iraq, Israel, Jordan, Kuwait, Lebanon, Oman, Qatar, Saudi Arabia, State of Palestine, Syrian Arab Republic, Turkey, United Arab Emirates, Yemen, Kazakhstan, Kyrgyzstan, Tajikistan, Turkmenistan, Uzbekistan

Southeast Asia - Brunei, Cambodia, Indonesia, Vietnam, Thailand, Philippines, Myanmar, Timor-Leste, Laos, Malaysia, India, Bhutan, Nepal, Maldives, Bangladesh, Sri Lanka, Pakistan East Asia: China, Hong Kong, Macao, Democratic People's Republic of Korea, Japan, Mongolia, Republic of Korea

Africa - Burundi, Comoros, Djibouti, Eritrea, Ethiopia, Kenya, Madagascar, Malawi, Mauritius, Mayotte, Mozambique, Réunion, Rwanda, Seychelles, Somalia, South Sudan, Uganda, United Republic of Tanzania, Zambia, Zimbabwe, Middle Africa, Angola, Cameroon, Central African Republic, Chad, Congo, Democratic Republic of the Congo, Equatorial Guinea, Gabon, Sao Tome and Principe, Algeria, Egypt, Libya, Morocco, Sudan, Tunisia, Western Sahara, Botswana, Lesotho, Namibia, South Africa, Swaziland, Western Africa, Benin, Burkina Faso, Cabo Verde, Cote d'Ivoire, Gambia, Ghana, Guinea, Guinea-Bissau, Liberia, Mali, Mauritania, Niger, Nigeria, Saint Helena, Senegal, Sierra Leone, Togo

Northern America - United States, Bermuda, Greenland, St. Pierre et Miquelon

Central America, Caribbean and Mexico - Mexico, Anguilla, Antigua and Barbuda, Aruba, Bahamas, Barbados, Bonaire Saint Eustatius and Saba, British Virgin Islands, Cayman Islands, Cuba, Curaçao, Dominica, Dominican Republic, Grenada, Guadeloupe, Haiti, Jamaica, Martinique, Montserrat, Puerto Rico, Saint-Barthélemy, Saint Kitts and Nevis, Saint Lucia, Saint Martin (French part), Saint Vincent and the Grenadines, Sint Maarten (Dutch part), Trinidad and Tobago, Turks and Caicos Islands, United States Virgin Islands, Belize, Costa Rica, El Salvador, Guatemala, Honduras, Nicaragua, Panama

South America - Argentina, Bolivia, Brazil, Chile, Colombia, Ecuador, Falkland Islands (Malvinas), French Guiana, Guyana, Paraguay, Peru, Suriname, Uruguay, Venezuela (Bolivarian Republic of)

Oceania - Australia, New Zealand, Norfolk Island, Melanesia, Fiji, New Caledonia, Papua New Guinea, Solomon Islands, Vanuatu, Micronesia, Guam, Kiribati, Marshall Islands, Micronesia (Federated States of), Nauru, Northern Mariana Islands, Palau, Polynesia, American Samoa, Cook Islands, French Polynesia, Niue, Pitcairn, Samoa, Tokelau, Tonga, Tuvalu, Wallis and Futuna Islands

© 2021 Quach BI et al. *JAMA Network Open.*

eTable 1. Comorbidities for Recent Immigrants and Long-standing Residents

Condition	Long-standing Residents (n = 354 194)	Recent Immigrants (n = 22 423)	Total (N = 376 617)
Acute myocardial infarction	10,521 (3.0%)	664 (3.0%)	11,185 (3.0%)
Arrhythmia	87,830 (24.8%)	4,122 (18.4%)	91,952 (24.4%)
Asthma	56,931 (16.1%)	3,558 (15.9%)	60,489 (16.1%)
Congestive heart failure	120,456 (34.0%)	6,736 (30.0%)	127,192 (33.8%)
Chronic obstructive pulmonary disease	90,556 (25.6%)	3,211 (14.3%)	93,767 (24.9%)
Cancer	142,109 (40.1%)	9,196 (41.0%)	151,305 (40.2%)
Coronary	138,643 (39.1%)	6,494 (29.0%)	145,137 (38.5%)
Dementia	108,094 (30.5%)	5,198 (23.2%)	113,292 (30.1%)
Diabetes	131,503 (37.1%)	9,793 (43.7%)	141,296 (37.5%)
Hypertension	272,702 (77.0%)	16,197 (72.2%)	288,899 (76.7%)
Inflammatory bowel disease (Crohn's or Colitis)	3,505 (1.0%)	98 (0.4%)	3,603 (1.0%)
Non-psychotic Mood and Anxiety Disorders	57,523 (16.2%)	3,595 (16.0%)	61,118 (16.2%)
Osteoarthritis	239,150 (67.5%)	12,393 (55.3%)	251,543 (66.8%)
Osteoporosis	42,367 (12.0%)	2,462 (11.0%)	44,829 (11.9%)
Renal Disease	100,175 (28.3%)	7,120 (31.8%)	107,295 (28.5%)
Rheumatoid Arthritis	11,927 (3.4%)	441 (2.0%)	12,368 (3.3%)
Stroke	62,567 (17.7%)	3,818 (17.0%)	66,385 (17.6%)
(Other) Mental Health Conditions	42,913 (12.1%)	2,668 (11.9%)	45,581 (12.1%)

© 2021 Quach BI et al. *JAMA Network Open.*

eTable 2. Location of Death for Recent Immigrants by Region of Origin and Time Since Immigration

**N = 22 423**	**Location of death**			
	**Subacute**	**Acute**	**Community**	**Long-term care**
**Region of Origin**				
Africa	87 (6.70%)	765 (58.89%)	385 (29.64%)	62 (4.77%)
Central America, Caribbean and Mexico	120 (7.00%)	979 (57.08%)	492 (28.69%)	124 (7.23%)
East Asia	365 (9.76%)	2123 (56.75%)	771 (20.61%)	482 (12.88%)
Eastern Europe	352 (10.46%)	1642 (48.80%)	1090 (32.39%)	281 (8.35%)
North America	37 (8.73%)	188 (44.34%)	151 (35.61%)	48 (11.32%)
Northern and Western Europe	92 (8.51%)	441 (40.80%)	358 (33.12%)	190 (17.58%)
Oceania	*	104 (59.77%)	*	*
Other	*	10 (58.82%)	*	*
South America	78 (6.45%)	696 (57.52%)	347 (28.68%)	89 (7.36%)
South-east Asia	482 (6.37%)	4691 (62.02%)	2050 (27.1%)	341 (4.51%)
Southern Europe	67 (8.12%)	439 (53.21%)	242 (29.33%)	77 (9.33%)
Western and Central Asia	94 (9.33%)	596 (59.13%)	263 (26.09%)	55 (5.46%)
**Time since Immigration (years)**				
0-2	59 (9.09%)	406 (62.56%)	166 (25.58%)	18 (2.77%)
3-5	105 (9.63%)	643 (58.99%)	312 (28.62%)	30 (2.75%)
6-10	185 (8.16%)	1318 (58.14%)	670 (29.55%)	94 (4.15%)
11-15	258 7.85%)	1852 (56.33%)	974 (29.62%)	204 (6.2%)
16-20	364 (7.61%)	2788 (58.29%)	1268 (26.51%)	363 (7.59%)

© 2021 Quach BI et al. *JAMA Network Open.*

21-25	513 (7.71%)	3729 (56.05%)	1765 (26.53%)	646 (9.71%)
26+	296 (8.02%)	1938 (52.48%)	1047 (28.35%)	412 (11.16%)

*Cells omitted due to potential risk for re-identification of small cell sizes.

© 2021 Quach BI et al. *JAMA Network Open.*

eTable 3. Negative Binomial Regression for Inpatient Care and Long-term Care Days in the Last 90 Days of Life for the Cohort of Recent Immigrants

**Exposure**	**Count of Inpatient (ICU and non-ICU) Days (95% CI)**		**Count of Long-term Care Days (95% CI)**	
	**Rate Ratio**	**P-value**	**Rate Ratio**	**P-value**
**Sex**				
Female	**Ref.**		**Ref.**	
Male	1.03 (0.99-1.07)	0.2064	0.68 (0.58-0.79)	<.0001
**Age at death, years**				
18-44	1.00 (0.91-1.08)	0.9235	0.25 (0.17-0.38)	<.0001
45-64	**Ref.**		**Ref.**	
65-84	0.86 (0.81-0.91)	<.0001	4.29 (3.49-5.26)	<.0001
85+	0.61 (0.57-0.65)	<.0001	10.11 (8.06-12.68)	<.0001
**Charlson comorbidity index**				
0	**Ref.**		**Ref.**	
1-2	4.40 (3.85-5.04)	<.0001	4.10 (2.37-7.09)	<.0001
3-4	6.25 (5.45-7.15)	<.0001	8.73 (5.07-15.00)	<.0001
5+	8.96 (7.81-10.27)	<.0001	13.11 (7.63-22.52)	<.0001
**Income quintile**				
First (lowest)	**Ref.**		**Ref.**	
Second	1.03 (0.98-1.09)	0.2219	0.79 (0.64-0.97)	0.0228
Third	0.99 (0.94-1.05)	0.7981	0.79 (0.64-0.99)	0.0361
Fourth	1.03 (0.97-1.10)	0.3102	0.92 (0.72-1.17)	0.4869
Fifth (highest)	0.99 (0.92-1.06)	0.6778	0.72 (0.55-0.94)	0.0141
**Community size**				
1 500 000+	1.00 (0.84-1.19)	0.9818	0.98 (0.53-1.85)	0.9621
500 000 - 1 499 999	0.98 (0.82-1.17)	0.8346	1.21 (0.63-2.34)	0.566
100 000 - 499 999	0.95 (0.79-1.13)	0.546	1.07 (0.56-2.06)	0.8316

© 2021 Quach BI et al. *JAMA Network Open.*

10 000 - 99 999	0.90 (0.71-1.14)	0.3743	2.03 (0.83-4.97)	0.1188
<10 000	**Ref.**		**Ref.**	
**Cause of death**				
Frailty	**Ref.**		**Ref.**	
Organ failure	0.97 (0.92-1.03)	0.387	0.35 (0.28-0.43)	<.0001
Terminal illness	1.43 (1.34-1.53)	<.0001	0.13 (0.09-0.15)	<.0001
Sudden death	0.57 (0.53-0.63)	<.0001	0.13 (0.09-0.18)	<.0001
**Other**	1.15 (1.04-1.29)	0.0099	0.50 (0.34-0.74)	0.0006
**Receipt of homecare in the last 90 days of life**				
No homecare	**Ref.**		**Ref.**	
Non-palliative homecare	1.36 (1.30-1.42)	<.0001	0.90 (0.76-1.05)	0.1845
Palliative homecare	0.80 (0.73-0.87)	<.0001	0.25 (0.18-0.36)	<.0001
**Receipt of palliative physician visit in the last 90 days of life**				
Yes	1.00 (0.95-1.06)	0.9098	1.15 (0.92-1.43)	0.2199
**Region of origin**				
Africa	1.35 (1.18-1.53)	<.0001	0.64 (0.40-1.02)	0.0609
Northern and Western Europe	**Ref.**		**Ref.**	
Western and Central Asia	1.25 (1.09-1.43)	0.0012	0.63 (0.38-1.02)	0.0618
Southern Europe	1.15 (1.00-1.33)	0.0504	0.63 (0.38-1.05)	0.074
South-east Asia	1.17 (1.05-1.30)	0.0042	0.61 (0.42-0.89)	0.0099
South America	1.11 (0.97-1.26)	0.1266	0.49 (0.31-0.79)	0.0033
Other	1.25 (0.60-2.58)	0.5511	3.56 (0.26-47.83)	0.3386
Oceania	1.05 (0.82-1.34)	0.6963	0.42 (0.17-1.02)	0.0554
North America	1.10 (0.92-1.30)	0.3005	0.54 (0.28-1.03)	0.0603
Eastern Europe	1.09 (0.98-1.22)	0.1248	0.78 (0.53-1.15)	0.212
East Asia	1.23 (1.10-1.38)	0.0003	0.88 (0.59-1.32)	0.5445
Central America, Caribbean and Mexico	1.22 (1.08-1.38)	0.0017	0.59 (0.38-0.91)	0.0169

© 2021 Quach BI et al. *JAMA Network Open.*

**Time since immigration**				
0-2	1.30 (1.14-1.48)	<.0001	0.67 (0.41-1.09)	0.109
3-5	1.19 (1.07-1.32)	0.0012	0.44 (0.30-0.65)	<.0001
6-10	1.21 (1.12-1.32)	<.0001	0.80 (0.58-1.11)	0.1805
11-15	1.11 (1.03-1.19)	0.0053	0.69 (0.53-0.91)	0.0072
16-20	1.06 (0.99-1.13)	0.0923	1.06 (0.83-1.36)	0.649
21-25	1.01 (0.95-1.07)	0.8452	1.01 (0.80-1.26)	0.9469
26+	**Ref.**		**Ref.**	

© 2021 Quach BI et al. *JAMA Network Open.*

eFigure 1. Mean Number of Days Spent in Each Place of Care in the Last 90 Days of Life Among Recent Immigrants by Region of Origin

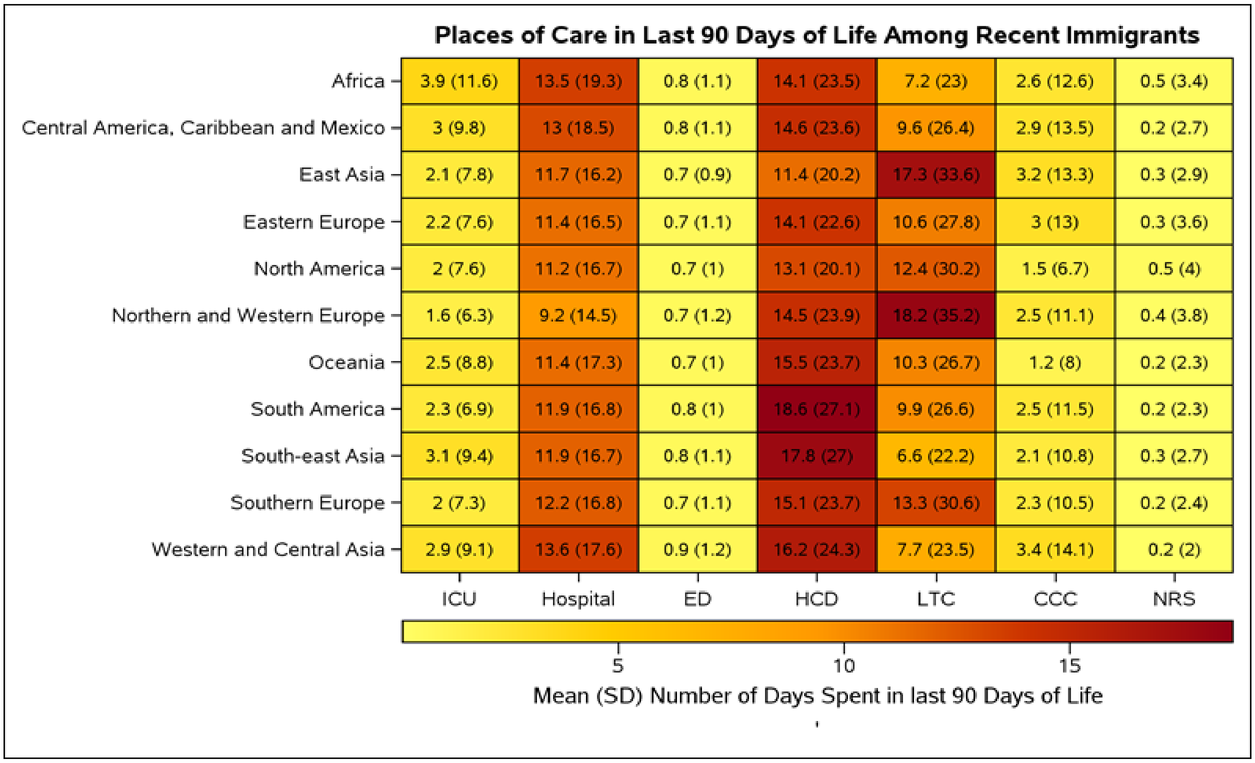

Footnote: Colour map of mean number of days spent in the last 90 days of life at each place of care among recent immigrants (N = 22 423). Comparisons are between recent immigrant decedents who originate from each region. Decedents with more yellow have a lower mean number of days spent in the last 90 days of life for the respective service; decedents with more red have a higher mean number of days spent in the last 90 days of life for the respective service. ICU: intensive-care unit hospitalization, Hospital: non-intensive-care unit hospitalization, ED: emergency department care, HCD: home care delivery service, LTC: long-term care service, CCC: complex continuing care service, NRS: rehabilitation service.

© 2021 Quach BI et al. *JAMA Network Open.*

eFigure 2. Comparison of Places of Care in the Last 90 Days of Life Among Recent Immigrants

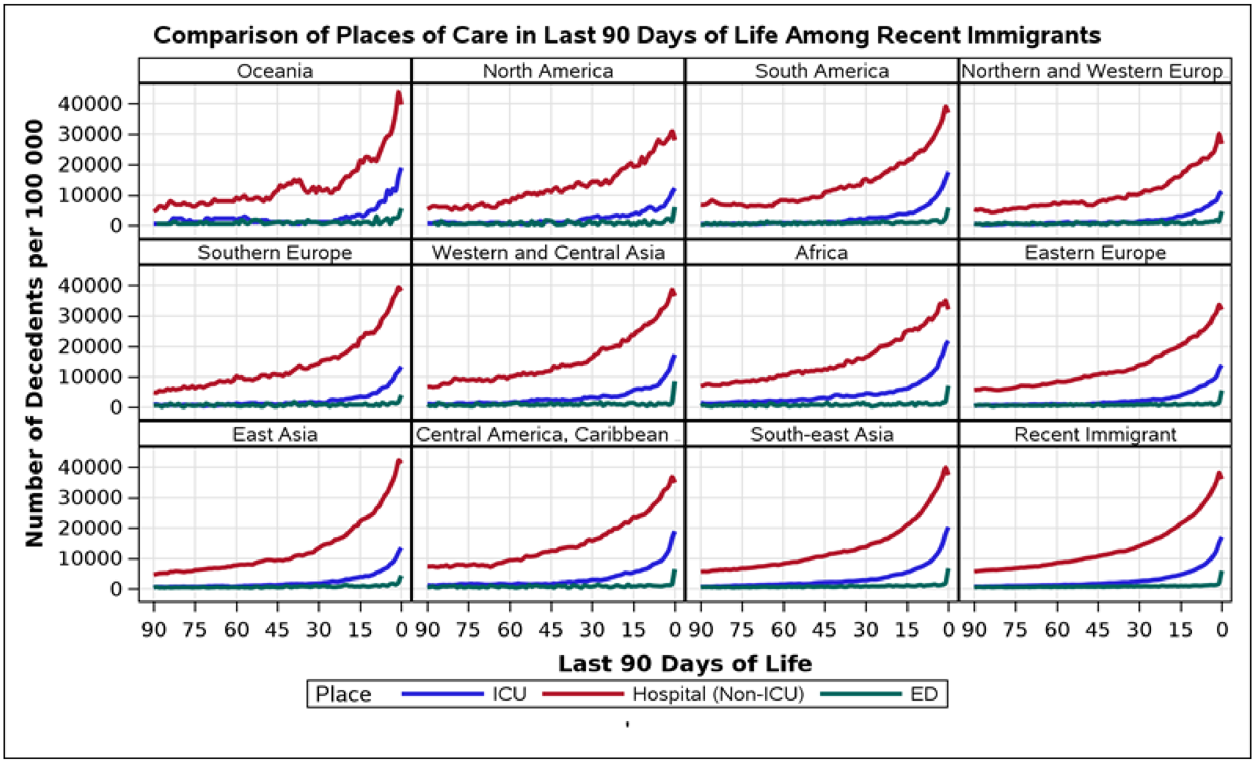

Footnote: 12-panel figure depicting the acute care service trends of recent immigrants (N = 22 423) in the last 90 days of life. Intensive-care unit hospitalization (ICU, blue), non-intensive-care unit hospitalization (Hospital Non-ICU, red), and emergency department care (ED, green) are projected along the X-axis representing the time interval preceding death in days and the Y-axis representing the number of decedents per 100 000 that received the respective service.

© 2021 Quach BI et al. *JAMA Network Open.*
